# The non-coding epitranscriptome in cancer

**DOI:** 10.1093/bfgp/elab003

**Published:** 2021-02-10

**Authors:** Valentina Miano, Azzurra Codino, Luca Pandolfini, Isaia Barbieri

**Keywords:** non-coding RNA, epitranscriptomics, RNA epigenetics, cancer, RNA modifications, RNA methylation

## Abstract

Post-synthesis modification of biomolecules is an efficient way of regulating and optimizing their functions. The human epitranscriptome includes a variety of more than 100 modifications known to exist in all RNA subtypes. Modifications of non-coding RNAs are particularly interesting since they can directly affect their structure, stability, interaction and function. Indeed, non-coding RNAs such as tRNA and rRNA are the most modified RNA species in eukaryotic cells. In the last 20 years, new functions of non-coding RNAs have been discovered and their involvement in human disease, including cancer, became clear. In this review, we will present the evidence connecting modifications of different non-coding RNA subtypes and their role in cancer.

## Introduction

More than 100 chemical modifications of RNA molecules have been discovered during the last several decades. These modifications are found in both prokaryotic and eukaryotic cells and have been observed on all RNA subtypes [1]. Despite their number and abundance, RNA modifications received relatively little attention until recently and consequently they remain generally poorly characterized. The reasons for this are both technical and historical. Firstly, techniques for the specific detection of RNA modifications (especially within the context of RNA sequence) were lacking and, even when available, were only able to quantify highly abundant modifications. Secondly, RNA biology received little attention in the past since, in the light of the central dogma of molecular biology, this biomolecule was seen as a mere intermediate of protein translation.

In the last two decades, the advent of next generation sequencing has allowed the development of new detection methods for RNA modifications. At the same time, the discovery of new RNA species and novel regulatory mechanisms mediated by RNA sparked renewed interest in RNA biology. In particular, the roles of newly identified non-coding RNAs (ncRNAs) such as micro RNAs (miRNAs) and long non-coding RNAs (lncRNAs) highlighted the scope of RNA biology both in physiology and disease.

These conditions paved the way for the development of the new field of epitranscriptomics. Several modifications were mapped to the transcriptome and new functions of RNA modifications were described. In particular, two primary RNA methylations, N-6-methyladenosine (m^6^A) [2, 3] and 5-methylcytosine (m^5^C) [4], were mapped to the transcriptome through next generation sequencing after specific enrichment or treatment. m^6^A-modified RNAs were enriched through RNA immunoprecipitation (RIP) with a specific antibody [2, 3] before sequencing. m^5^C-carrying RNAs were firstly enriched through m^5^C-specific RIP and then subjected to bisulphite treatment [4] in order to detect m^5^C at base resolution. The enzymes responsible for these modifications, the METTL3/METTL14 complex for m^6^A [5] and the NSUN family for m^5^C [6], were discovered and characterized. Furthermore, the functions of these modifications were the first to be identified. m^6^A was shown to promote degradation of cellular mRNAs [7, 8], while m^5^C was shown to increase the stability of transfer (t)RNAs [9, 10] and to regulate the processing of the *VAULT* ncRNAs (*vtRNA*) [11]. Finally, the identification of the demethylases FTO [12] and ALKBH5 [13] as m^6^A erasers and the discovery of the YTH protein family as specific m^6^A readers [14] showed that RNA epigenetic modifications, similarly to chromatin modifications, can be dynamic and are capable of triggering specific downstream molecular pathways. More recently, other modifications such as pseudouridine (Ψ) [15], N1-methyladenosine (m^1^A) [16, 17] and 7-methylguanosine (m^7^G) [18–20] were mapped and characterized by using a combination of chemical reactivity methods and specific antibodies.

Depending on their position within nucleotides, RNA modifications can affect the pairing of RNA bases, therefore impacting the secondary structures and physiological functions of RNAs. For example, the deamination of adenosines to inosines (A-to-I), mediated by ADAR1 and ADAR2, converts base pairing from A-T to I-G [21]. RNA editing is used by eukaryotic cells to prevent the formation of dsRNA derived from transposable elements, to change the target pool of miRNAs or to alter the coding sequence of mRNAs [21]. Other, non-conventional base pairings such as Hoogsteen pairing can also be affected by RNA modification such as m^7^G or Ψ [22].

The general functions of RNA modifications were thoroughly reviewed by Roundtree and colleagues [1]. Recently, their role in the context of human disease and in particular in cancer, has come under the spotlight. An increasing amount of evidence now not only shows that RNA-modifying enzymes can affect the phenotype of cancer cells [23] but also suggests that they may represent viable molecular targets for new anticancer treatments [24].

While mRNA is an intermediate of gene expression, ncRNAs are effector molecules and their functions mostly rely on a correct structural folding and activity. Since modifications can directly affect RNA stability and structure, they might be particularly important for proper ncRNAs functioning.

Furthermore, ncRNA covalent marks are in many cases needed to fine-tune RNA function and are often dispensable for normal physiology, but their mis-regulation can promote tumour growth. Consequently, the enzymes responsible for such marks represent ideal candidates to specifically target cancer cells. In this review, we will focus on the modifications occurring on the different types of ncRNAs and discuss their role in cancer.

### Ribosomal RNA

Ribosomes are molecular complexes composed of 80 protein subunits and 4 different ribosomal RNAs (rRNAs), *28S*, *18S*, *5S* and *5.8S* [25]. Three out of four are transcribed by RNA polymerase I (POLI) as a single precursor RNA, while *5S* is transcribed by RNA polymerase III (POL3). In the nucleolus, rRNAs are processed co-transcriptionally by a large number of protein complexes [25]. rRNA modifications are mediated by two types of enzymatic complexes, (1) RNA-guided modifiers, which require the action of small nucleolar RNAs (snoRNAs) to identify the target nucleotides and (2) stand-alone enzymes [26]. They can be either deposited co-transcriptionally on unprocessed rRNA or post-transcriptionally on mature rRNAs and are required for the correct assembly of functional ribosomes [26]. rRNA modifications are generally highly conserved from yeast to human cells and are more frequent within functionally important regions of rRNA molecules [26].

**Figure 1:**
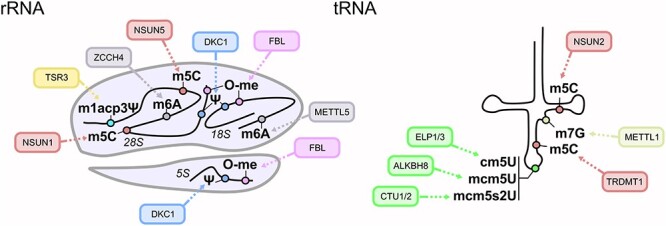
rRNA and tRNA modifications involved in cancer. Figure depicts RNA modifications (circles) on ribosomal (rRNA, left panel) and transfer RNA (tRNA, right panel) that have been connected to cancer. The enzymes responsible for their deposition are indicated in the balloons. m^1^acp^3^Ψ: 1-methyl-3-a-amino-a-carboxyl-propyl pseudouridine; m^6^A: 6-methyladenosine; m^5^C: 5-methylcytosine; O-me: 2’O-methylation; Ψ: pseudouridine; m^7^G: 7-methylguanosine; cm^5^U: 5- carboxymethyluridine; mcm^5^U: 5- methoxycarbonylmethyluridine; mcm^5^s^2^U: 5-methoxycarbonylmethyl-2-thiouridine.

The most abundant modification on rRNAs is the 2’-O-methylation of ribose residues, mediated by a complex including C/D box snoRNAs [27] and the methyltransferase fibrillarin (FBL) [28]. This modification is found on tens of nucleosides within rRNAs and occurs on all four nucleotides (Figure 1). More than 20 different snoRNAs exist in human cells and each one is responsible for a subset of O-methylation events on rRNAs [29]. Mechanistically, 2’-O-methylation stabilizes rRNA structure and ensures fidelity of translation in physiological conditions [26]. Despite this, aberrantly elevated levels of rRNA 2’-O-methylation are associated with impaired translation fidelity in cancer cells. For example, FBL overexpression is associated with global upregulation of rRNA 2’-O-methylation, which in turn causes stop codon bypass and amino acid misincorporation [30]. Additionally, this modification seems to be required for IRES-dependent translational initiation [31]. Indeed, FBL overexpression in cancer increases IRES-dependent translation of key oncogenes such as *c-MYC* and *VEGFA* [30]. Importantly, FBL is highly expressed in human breast and prostate cancer and its expression positively correlates with poor prognosis [30, 32] (Table 1).

**Table 1:** Roles of Ribosomal RNA and Transfer RNA modifications in cancer

**Ribosomal RNA**			
***Modification***	***Enzyme/reader***	***Involvement in cancer biology***	***Reference***
**Nm**	**FBL**	**FBL overexpression suppresses p53 expression in human breast and prostate cancers**	**[30,32]**
**Nm**	***SNORD50***	**Low levels SNORD50 increase c-Myc oncogene expression in human colorectal cancer**	**[35]**
**Ψ**	**DKC1**	**Loss of DKC1 affects the translation of VEGF and p53 in human head and heck squamous cell carcinoma and pituitary cancer**	**[42,43]**
**m** ^**1**^ **acp** ^**3**^ **Ψ**	**TSR3**	**rRNA mutations causes loss of modification in human colorectal cancer**	**[46]**
**m** ^**6**^ **A**	**ZCCH4**	**ZCCH4 overexpression in human hepatocellular and liver cancer**	**[47]**
**m** ^**5**^ **C**	**NSUN5**	**Loss of NSUN5 and m** ^**5**^**C in human glioma**	**[51]**
**Transfer RNA**			
***Modification***	***Enzyme/reader***	***Involvement in cancer biology***	***Reference***
**cm** ^**5**^ **U**	**ELP1, ELP3**	**Overexpression of ELP1/3 mediates metabolic switch and BRAF inhibitors resistance in melanoma**	**[61]**
**cm** ^**5**^ **U**	**ELP3**	**Overexpression of ELP3 promotes translation of pro-metastatic genes in breast cancer**	**[63]**
**cm** ^**5**^ **U**	**ELP3**	**Overexpression of ELP3 and tRNA cm** ^**5**^**U modification promote maintenance of colorectal cancer stem cells**	**[64]**
**mcm** ^**5**^ **s** ^**2**^ **U**	**CTU2**	**Overexpression of CTU2 mediates metabolic switch and BRAF inhibitors resistance in melanoma**	**[61]**
**mcm** ^**5**^ **s** ^**2**^ **U**	**CTU1, CUT2**	**Overexpression of CTU1/2 promotes translation of pro-metastatic genes in breast cancer.**	**[63]**

m^1^acp^3^Ψ, 1-methyl-3-a-amino-acarboxyl-propyl pseudouridine; m^6^A, 6-methyladenosine; m^5^C, 5-methylcytosine; Nm, 2'O-methylation; ψ, pseudouridine; cm^5^U, 5-carboxymethyluridine; mcm^5^s^2^U, 5-methoxycarbonylmethyl-2-thiouridine.

Indeed, FBL expression negatively correlates with p53 expression in breast and prostate cancer. Marcel and colleagues showed that p53 can directly regulate FBL expression through direct binding to the *FBL* promoter. In turn, high levels of FBL can regulate p53 activation in response to cellular stress and suppress its translation [30, 32]. In this scenario, *p53* mutations de-repress FBL transcription and promote tumour progression. On the other hand, overexpression of FBL could start a feedback loop to repress the p53 tumour suppressive pathway in breast cancers expressing wild-typep53.

While global FBL overexpression appears to have an oncogenic effect, it was also shown that selected snoRNAs have specific oncogenic functions in AML-ETO1-mediated leukemic transformation [33]. In this subtype of leukaemia, high expression of *SNORD34*, *SNORD35* and *SNORD43* is necessary for the establishment of leukemic blasts without affecting general translation levels [33]. In contrast, several studies showed that *SNORD50*, mediating the modification on *28S*-C2848 and *28S*-G2863, could be tumour suppressive in human cancers [34], including colon cancer [35], prostate cancer [36], breast cancer [37] and B-cell lymphoma [38]. Molecularly, colon cancer cells expressing low levels of *SNORD50* showed increased levels of IRES-dependent translation of *c-MYC* [35] (Table 1). Taken together, these studies show a complex scenario, where specific subsets of rRNA modifications can have opposite, tumour type-dependent effects on cancer progression.

The second most abundant modification on rRNA is the isomerization of uridine residues into pseudouridines (Ψ). This modification is present in over 100 sites throughout all rRNA subunits [39]. Ψ deposition is mediated by a complex including H-ACA box snoRNAs and the uridine isomerase DKC1 (Figure 1). Target identification is mediated by snoRNAs, similarly to the deposition of 2’-O-methylation [40]. Mutations inactivating the catalytic activity of DKC1 are responsible for dyskeratosis congenita, a complex syndrome characterized by bone marrow failure and predisposition to cancer [41]. Initially, since DKC1 can modify both rRNAs and telomerase RNA, the main mechanism for the increased onset of cancer in dyskeratosis congenita patients was unclear. Subsequently, it was shown that the phenotype of a mouse model carrying DKC1-inactivating mutations found in human dyskeratosis congenita was dependent on the decreased modification of *28S* rRNA and the aberrant translation of oncogenic and tumour-suppressive mRNAs, including *VEGF* and *p53* [42] (Table 1). DKC1 was also shown to be a tumour suppressor in pituitary tumorigenesis: in this cancer type, lack of rRNA pseudouridylation causes a decrease in IRES-dependent expression of the tumour suppressor p27 [43] (Table 1). Other studies showed that DKC1 can be overexpressed in lung [44] and prostate cancer [45]. These studies show that Ψ modifications on rRNA are disproportionally important for translation of both tumour-suppressive and pro-oncogenic factors.

Secondary hyper-modification of Ψ1248 within *18S,* mediated by TSR3, generates 1-methyl-3-a-amino-a-carboxyl-propyl pseudouridine (m^1^acp^3^Ψ) (Figure 1), which is lost in several different types of human cancers [46]. In particular, 45% of colorectal carcinomas show decreased levels of this modification. However, TSR3 is not mutated or downregulated in tumour samples. Interestingly, loss of this modification occurs through mutations of rRNAs, generating cancer-specific ‘oncoribosomes’ [46] (Table 1). The molecular mechanism by which mutated ribosomes promote cancer growth is still unknown.

Additionally, there are less abundant modifications found in rRNA, such as m^6^A and m^5^C. The former is present on one residue in 28*S* (A4220) [47] and one in *18S* (A1832) [48], while the latter is found on two residues in *28S* rRNA (C3761, C4413) [6].

m^6^A on *18S* is catalysed by METTL5 [48], whilst on *28S* RNA, it is catalysed by ZCCH4 [47] (Figure 1). There is little knowledge on the function of these enzymes in the context of cancer, but ZCCH4 is overexpressed in hepatocellular cancer cells and human liver cancer [47]. It seems that the translation of a subset of mRNAs involved in membrane trafficking may be particularly affected by ZCCH4 downregulation and loss of m^6^A on 28*S* [47] (Table 1).

Modification of 28*S* rRNA with m^5^C is mediated by NSUN1 on C4413, and by NSUN5 on C3761 [6] (Figure 1). NSUN1 is associated with high proliferation levels and correlates with poor prognosis in lung [49] and prostate cancer [50]. Despite this, it is not clear whether it has a specific function in transformed cells or it is just associated with high cell cycle rates. Recently, it has been shown that NSUN5 is lost from a significant subgroup of human gliomas [51] (Table 1). Loss of m^5^C on C3761 decreases mRNA translation output globally [51]. The authors suggested that NSUN5 loss contributes to the protection of glioma cells from stress conditions. Despite being a tumour suppressor, loss of NSUN5 correlates with good prognosis in gliomas [51]; while NSUN5 helps to safeguard against stress conditions in the early stages of tumorigenesis, it is likely that its loss limits the proliferation potential of fully transformed glioma cells.

Taken together, these studies show that the epigenetic modifications of rRNAs, one of the most fundamental RNA subtypes in eukaryotic cells, are widely exploited by cancer cells. Notably, the regulation of cap-independent translation of key oncogenes and tumour suppressors as well as the general repression of translation in stress conditions are a common theme of the current experimental evidence.

### Transfer RNA

Transfer RNAs are short, highly structured RNA molecules fundamental for protein translation. They are transcribed by RNA polymerase III and undergo a maturation process in the nucleus [52], before translocating to the cytoplasm. Abnormal expression of tRNAs was observed in several cancer types [53], where it increases translation levels of specific oncogenic proteins [54]. Furthermore, both precursor and mature tRNA can be cleaved to form tRNA derivatives such as tRNA-derived stress-induced RNAs (tiRNAs), tRNA-derived fragments (tRFs) and tRNA-derived small RNAs (tsRNAs) [53]. These tRNA derivatives were shown to affect gene expression by controlling RNA stability [55] and translation [56]. Furthermore, specific tRNA fragments are overexpressed in rapidly dividing cancer cells [57] and can be dysregulated during cancer progression [58].

tRNAs are the most heavily modified RNA type in eukaryotic cells. They are decorated with a wide array of modifications, which have a variety of functions [52]. Generally, modifications within the anticodon are required for decoding: in particular, modifications of the wobble position ensure accurate decoding during translation and allow the pairing between mRNA codons and non-perfectly complementary tRNA anticodons reducing the variety of tRNAs required for correct translation [52]. On the other hand, modifications outside the anticodon positions are usually required to maintain the stability of tRNAs and prevent the generation of tRNA derivatives [52]. Similar to rRNAs, modification of tRNAs can have specific functions in cancer, without affecting the general translational output of normal cells.

Modifications of the U34 wobble position of a subset of tRNAs (*tRNA^Lys^UUU*, *tRNA^Glu^UUC*, *tRNA^Gln^UUG*, *tRNA^Gly^UCC* and *tRNA^Arg^UCU*) are necessary for accurate translation (Figure 1). Modifications of U34 are deposited in a sequential way: firstly, the elongator complex (ELP1/3) catalyses the conversion of uridine into 5-carboxymethyluridine (cm^5^U) [59] (Figure 1). Next cm^5^U is converted into 5-methoxycarbonylmethyluridine (mcm^5^U) by ALKBH8[59]. Finally, the thiolase enzymes CTU1 and CTU2 convert mcm^5^U into 5-methoxycarbonylmethyl-2-thiouridine (mcm^5^s^2^U) [59]. This last step only occurs in *tRNA^Lys^UUU*, *tRNA^Glu^UUC* and *tRNA^Gln^UUG*. Despite being catalysed in a tightly controlled manner and being necessary for accurate transcription, depletion of the enzymes responsible for U34 modifications is not generally lethal in yeast or normal human cells [60]. Strikingly though, it was recently shown that cancer cells particularly depend upon them to maintain translational levels of key oncogenes.

The enzymatic subunits of the elongator complex ELP1 and ELP3 and the thiolase CTU2 are overexpressed in human melanoma, particularly in melanomas carrying the BRAFV600E mutation [61] (Table 1). High levels of the U34 modifying enzymes are required to maintain the expression of proteins responsible for the metabolic switch toward glycolysis (such as HIF1α) observed in melanoma cells [61]. BRAF inhibitors are an approved therapy for BRAFV600E melanoma [62]. Despite this, response to treatment is often short-lived since cancer cells develop resistance to BRAF inhibition [61]. Importantly, downregulation of U34 enzymes in resistant melanoma cells can rescue the response to small molecule BRAF inhibitors [61]. Clinically, this is particularly important since the development of inhibitors specifically blocking the activity of the U34 enzymes could be used to increase sensitivity to BRAF inhibitors and prevent resistance.

ELP3, CTU1 and CTU2 are also overexpressed in breast cancer (Table 1), where they maintain high translation levels of the RNA-binding protein DEK1. This in turn promotes translation of the transcription factor LEF1, thereby upregulating pro-metastatic genes [63] (Table 1).

ELP3 is also overexpressed in colorectal cancer and it is required for tumour initiation in a WNT-driven colorectal cancer mouse model [64] (Table 1). In this model, ELP3 transcription is directly increased by WNT, and ELP3-mediated modification of U34 tRNA increases the translation of SOX9 [64], which in turn maintains colorectal cancer stem cells. Taken together these data show that pharmacological inhibition of U34-modifying enzymes may represent a viable approach for the generation of new cancer therapies.

m^5^C is found on a subgroup of tRNAs at several positions and is mediated by NSUN2 [65], DNMT2 [66] and NSUN6 [67] (Figure 1). Its function is to protect tRNAs from degradation [9, 68] and to avoid the production of tRNA fragments, functional tRNA derivatives capable of acting as miRNAs and regulating gene expression [53]. NSUN2 is upregulated by c-MYC and is overexpressed in breast cancer and head and neck carcinomas [69].

Similar to m^5^C, m^7^G protects tRNAs from degradation [20]. The writer of this modification is the METTL1/WDR4 complex, in which METTL1 is the active catalytic subunit [70] (Figure 1). METTL1 is overexpressed in hepatocellular carcinoma [71] and glioblastoma [72]. High levels of m^7^G are likely required to maintain a high level of translation in proliferating cancer cells. It is not clear whether the activity of m^5^C and/or m^7^G enzymes on tRNAs directly contributes to tumorigenesis or whether it is just required to sustain high levels of cell proliferation. Interestingly, overexpression of tRNA m^5^C and m^7^G enzymes increases sensitivity of human cells to 5-fluorouracil [73].

Crucially, all of the above studies were focused on the overexpression of NSUN2 and METTL1 without reporting the overall extent of m^5^C and m^7^G in cancer cells. Therefore, it is possible that their role in cancer may be independent from their catalytic activity. Future studies addressing this possibility are required to better elucidate the role of m^5^C and m^7^G tRNA modification in cancer.

Finally, 3-methylcytidine (m^3^C) occurs at different positions in several tRNAs and its deposition is catalysed by two tRNA-specific enzymes, METTL2B and METTL6 [74]. Although more investigation is required to reveal the distribution and dynamics of m^3^C insertion in mammalian tRNAs, it was recently demonstrated that m^3^C is a pre-requisite for C-to-U deamination in protozoans [75]. METTL6 catalyses m^3^C at C32 in specific tRNA^Ser^ isoacceptors. Depletion of METTL6 in hepatocellular cancer cells (HCCs) affects translation of mRNAs related to cell proliferation and growth [76]. Importantly, it was shown that this effect is dependent on its catalytic activity. Moreover, METTL6 was found amplified in different cancer cells and its amplification predicts a worse outcome for patients, whereas its low expression correlates with increased survival of HCC patients [76].

Thus, tRNAs are highly decorated with a variety of modifications. Unexpectedly, many epitranscriptomic marks occur only in subsets of tRNAs and they can become specifically dysregulated in cancer. Depending on codon usage, each specific modification is required for translation of a subset of mRNAs. Taken together, these studies show that cancer cells may be ‘addicted’ to specific tRNA modifications, dispensable for non-transformed cells. Consequently, inhibition of tRNA-modifying enzymes may represent a new, unexpected therapeutic approach for cancer.

### MicroRNA

MicroRNAs are ~22 nucleotide RNA molecules that are produced by a complex biosynthetic pathway [77]. This process is regulated at many levels by post-transcriptional nucleotide modifications, which are able to regulate either the RNA–RNA or RNA–protein interactions required for miRNA maturation and activity. Mechanistically, miRNA modifications act by tuning the RNA biophysical properties and/or altering their affinities for the biosynthesis/effector machinery, which often result in profound biological consequences.

**Figure 2:**
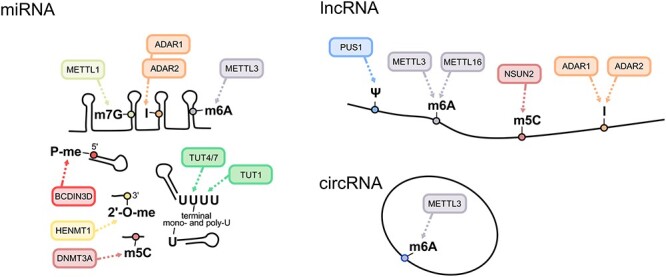
miRNA, lncRNA and circRNA modifications involved in cancer. Figure depicts RNA modifications (circles) on microRNAs (miRNA, left panel), long non-coding RNAs (lncRNA, top right panel) and circular RNAs (circRNAs, bottom right panel) that have been linked to cancer. The enzymes responsible for their deposition are indicated in the balloons. m^7^G: 7-methylguanosine; I: inosine; m^6^A: 6-methyladenosine; P-me: 5’-methylphosphate; 2’-O-me: 2’ O-methylation; m^5^C: 5- methylcytosine; Ψ: pseudouridine.

Non-templated nucleotide addition to the 3' end of miRNAs impacts the fate of miRNAs and plays important biological functions [78]. Indeed, uridylation of miRNA tails mediated by the terminal uridyltransferases TUT1, TUT4 and TUT7 has been implicated in a variety of cancers [78, 79]. The extent of uridylation on miRNA tails causes different outcomes on miRNA stability and fate (Figure 2). Poly-uridylation of *pre-let-7* miRNA, induced by the LIN28A and LIN28B proteins, impairs *let-7* biogenesis by hindering Dicer processing [80] (Figure 2). Mechanistically, LIN28-mediated repression of *let-7* is achieved through the recruitment of the TUT4 and TUT7 enzymes to *pre-let-7*, which results in *pre-let-7* poly-uridylation and its subsequent degradation [81, 82]. Importantly, the LIN28/*let-7* pathway is involved in cancer progression by regulating a broad range of processes including cell proliferation, metastasis, drug resistance and metabolism [79]. For instance, the Wnt-β-catenin pathway enhances LIN28 levels causing a decrease in mature *let-7* miRNA, thus driving proliferation of breast cancer stem cells [83]. Moreover, LIN28B supports head and neck cancer cell lines’ growth through the modulation of the insulin growth factor (IGF) pathway [84]. Downregulation of the TUT1 enzyme, which poly-uridylates miRNAs, increases osteosarcoma cell proliferation and invasiveness through the modulation of *miR-24* and *29a* expression levels [85].

Notably, a specific class of pre-miRNAs with a short (1-nucleotide) 3' overhang, including most *let-7* family members, are subject to TUT2/4/7-dependent mono-uridylation in differentiated cells [86]. However, in contrast to poly-uridylation, mono-uridylation of miRNAs does not trigger their degradation. Instead, this modification allows the extension of miRNA 3' ends, which is required for efficient Dicer processing and miRNA maturation [86].

Overall, poly- and mono-uridylation of miRNA precursors represents a common mechanism to regulate miRNA levels and can contribute to human tumorigenic processes.

miRNAs can also undergo m^6^A methylation, deposited by METTL3 on miRNA precursors. METTL3/METTL14-dependent m^6^A methylation of primary miRNAs improves the recognition and binding of pri-miRNAs to DGCR8, thereby enhancing miRNA processing and maturation [87] (Figure 2).

METTL14 has also been shown to be involved in the regulation of miRNAs processing in the context of hepatocellular carcinoma (HCC), where it prevents cancer progression [88]. Human liver cancers displaying low levels of both METTL14 and m^6^A-modification correspond to high metastatic potential and poor patient survival [88] (Table 2). Mechanistically, METTL14 interacts with DGCR8 and its depletion causes accumulation of the unprocessed *pri-miR-126*, a metastasis-suppressing miRNA [88]. Moreover, overexpression of METTL14 increases the levels of m^6^A-modified *pri-miR-126* and the amount of *pri-miR-126* associated with DGCR8 [88]. Thus, these data suggest that METTL14 controls m^6^A modification of *pri-miR-126* either directly or indirectly, to enhance its processing and antagonize the metastatic potential of HCC cells. Furthermore, the same authors report that METTL14 levels are reduced in breast cancer and they are associated with low survival rates, suggesting that METTL14 could potentially regulate m^6^A modification of miRNAs also in this type of tumour [88]. However, in these reports, it is not clear whether the DGCR8/METTL14 interaction is METTL3 dependent and what is the role of the methyltransferase activity in controlling the proposed pathological mechanisms.

**Table 2:** Roles of MicroRNA and Long non-coding RNA modifications in cancer

**MicroRNA**				
***Target***	***Modification***	***Enzyme***	***Involvement in cancer biology***	***References***
**pre-let-7**	**poly-U**	**TUT1,TUT4/7**	**Overexpression of TUT1 and TUT4/7 promotes degradation of let7 in breast cancer and head and neck carcinoma**	**[84,85]**
**pri-miR-126**	**m** ^**6**^ **A**	**METTL14**	**METTL14 and m** ^**6**^ **A promote maturation of tumour suppressor miRNAs in hepatocellular carcinoma**	**[89]**
**miR-17-5p**	**m** ^**6**^ **A**	**METTL3/METTL14**	**Methylated microRNA as biomarker for pancreatic cancer**	**[90]**
**pre-let-7**	**A-to-I**	**ADAR1**	**A-to-I editing of let-7 promotes cancer stem cells renewal in chronic myelogenous leukaemia**	**[94]**
**miR-378a-3p, miR-455-5p**	**A-to-I**	**ADAR1, ADAR2**	**A-to-I editing of miRNA seed sequences prevents progression and metastasis of human melanoma**	**[95,96]**
**miR-21, miR221/222, miR-589-3p**	**A-to-I**	**ADAR2**	**Loss of ADAR2 and miRNA editing promotes progression of human glioblastoma**	**[97,98]**
**Long non-coding RNA**				
***Target***	***modification***	***Enzyme***	***Involvement in cancer biology***	***References***
**XIST**	**m** ^**6**^ **A**	**METTL3/METTL14**	**Loss of METTL14 stabilizes XIST transcript supporting proliferation of human colorectal cancer**	**[107]**
**RP11**	**m** ^**6**^ **A**	**METTL3**	**Overexpression of METTL3 and RP11 modification stabilizes pro-tumourigenic transcription factors in colorectal cancer**	**[111]**
**NMR (LINC01672)**	**m** ^**5**^ **C**	**NSUN2**	**Overexpression of NSUN2 and modified NMR upregulates pro-metastatic factors in human oesophageal carcinoma**	**[122]**
**dsAlu transcripts**	**A-to-I**	**ADAR1**	**A-to-I editing of Alu sequences induces resistance to immunotherapy in human metastatic melanoma**	**[127]**

I, inosine; m^6^A, 6-methyladenosine; m^5^C, 5-methylcytosine.

METTL3 and METTL14 are highly expressed in gastrointestinal cancer tissues and, consistent with this finding, a subset of miRNAs exhibits high m^6^A levels in similar types of cancer [89]. In particular, m^6^A methylation of *miR-17-5p* is specifically detected in tumour biopsies from pancreatic cancer patients; therefore, methylated-*miR-17-5p* was suggested as a biomarker for early-stage pancreatic cancer [89] (Table 2).

miRNA modifications also include m^7^G at internal positions (Figure 2), as shown by the presence of this modification on a specific group of regulatory miRNAs, which suppress cancer cell migration [19]. In A549 lung cancer cell line, high levels of METTL1 mediate m^7^G deposition on a subset of miRNAs (Figure 2). Despite this, genome-wide mapping of m^7^G in a different cellular model failed to detect the modification on RNAs other than tRNAs [90], possibly due to different assay sensitivity and/or inherent biological differences.

Loss of METTL1 catalytic activity in A549 cells leads to the upregulation of migratory mRNAs containing *let-7* target sequence, such as HMGA2 [19]. Indeed, METTL1 methylates *let-7* pri-miRs at specific positions overlapping the 5’ site of DROSHA cleavage and spanning G-rich sequences [19]. Thus, METTL1-mediated m^7^G modification of *let-7* pri-miR counteracts the formation of non-canonical secondary structures in *let-7* pri-miR, thereby favouring its processing and enhancing miRNA-mediated repression of migratory mRNAs [19]. The position of m^7^G on mature *let-7* at single nucleotide resolution was determined through mass spectrometry fingerprinting [19]. Despite this, it cannot be excluded that the methylation pattern observed could originate from an O’-methylated *rRNA* fragment [90]. Improved, more sensitive methods of m^7^G detection will be required to highlight the relevance of this modification on miRNA and its importance in cancer.

5-Methylcytosine was also identified in miRNAs [91], where it is deposited by the DNMT3A/AGO4 [91] complex (Figure 2) and exerts important regulatory functions. For example, m^5^C in *miR-181a-5p* alters its ability to repress its mRNA targets [91]. Remarkably, the cytosine-methylation status of *miR-181a-5p* can be used for the prognosis of glioblastoma patients, as high methylation levels correlate with low survival rates [91] .

miRNAs are also subject to ADAR1- and ADAR2-mediated editing, which involves deamination of adenosine to inosine [21]. A-to-I-editing of miRNAs can impact either their biogenesis or their repertoire of mRNA targets, with important biological consequences such as tumour suppression or cell growth [92] (Figure 2). Notably, ADAR1-dependent editing of *let-7* pri-miRNA impairs *let-7* maturation, thereby promoting leukaemia stem cell self-renewal [93] (Table 2). ADAR1 act as a tumour suppressor in melanoma where it is downregulated [94]. In normal melanocytes, ADAR1 edits *miR-378-3p* sequence to target the *PARNA* oncogene [94] (Table 2). Similarly, loss of ADAR2-mediated editing within *miR-455-5p* seed sequence alters the recognition of *miR-455-5p* mRNA targets, favouring melanoma progression and metastasis [95] (Table 2).

Maintaining physiological levels of A-to-I miRNA editing is also critical to counteract glioblastoma proliferation and migration [96, 97]. Indeed, ADAR2-mediated editing of the *onco-miR-221/222* and *-21* precursors represses the respective mature miRNAs in normal brains and hinders glioblastoma growth [97] (Table 2). Furthermore, A-to-I editing of *miR-589-3p* seed sequence acts as a molecular switch to control glioblastoma invasiveness [96] (Table 2). Under physiological conditions, ADAR2 edits the *miR-589-3p*, which, in turn, targets the tumour suppressor *PCDH* mRNA [96]. However, upon ADAR2 loss, the unedited *miR-589-3p* targets the *ADAM12* mRNA, which promotes glioblastoma progression [96]. Although it is clear that A-to-I editing is a common mechanism for redirecting miRNA targeting in human glioblastoma, the clinical relevance of this process is still poorly understood.

While miRNA precursors and mature miRNAs are not normally capped, cap analogous modifications of 5’ miRNA terminal moieties have been reported [98]. O-Methylation of 5' monophosphate (5' P-me) of *pre-miR-145* is catalysed by BCDIN3D methyltransferase and was shown to interfere with Dicer processing and *pre-miR-145* maturation [98] (Figure 2). Importantly, BCDIN3D depletion in breast cancer cells increases *miRNA-145* mature isoform and reduces cell invasiveness [98]. More recently, BCDIN3D was shown to methylate cytoplasmic *tRNA^His^* [99] and to regulate the formation of tRNA fragments [100]. However, further studies are needed to understand the relative contribution of BCDIN3D tRNA methylation activity to breast cancer.

Another type of miRNA terminal methylation is the 2'-O-methylation on the last ribose of the molecule (3'-terminal 2'O-me) [101]. In particular, 3'-terminal 2'O-me of *miR-21-5p* mediated by the HENMT1 methyltransferase protects miRNA from 3' to 5' exoribonucleolytic cleavage and strengthens AGO2 binding [101] (Figure 2). Notably, 3'-terminal 2'O-me of *miR-21-5p* is detected in lung cancer tissues but not in healthy ones [101], underlining the biological relevance of terminal miRNAs methylation *in vivo*.

Future work will be required to confirm and explore in deeper molecular detail the mechanisms described in the previous reports. Most RNA modifications and their effects appear to be highly context dependent, possibly due to the fact that they impinge on different RNA targets and downstream pathways. Therefore, especially for miRNAs, it will be necessary to gain a better understanding of the molecular factors determining the specificity of RNA-modifying activity (e.g. RNA structural features and motifs, protein interactors and enzyme/cofactor expression).

Overall, miRNA covalent modifications may represent a post-transcriptional phenomenon to establish and finely modulate a wide range of cellular programmes in different cell types. Thus, a thorough characterization of miRNA modifications and their related catalytic machinery may help to dissect the molecular basis of cancer. Furthermore, the possibility that miRNAs could be specifically modified in selected human malignancies could be leveraged to set up more robust cancer biomarkers.

## Long non-coding RNA

Long ncRNAs are heterogeneous RNA transcripts longer than 200 nucleotides that are not translated into protein. They include long-intergenic RNAs (lincRNAs), antisense transcripts to mRNAs, enhancer RNAs (eRNAs) and RNAs deriving from transcription of transposable elements [102]. LncRNAs are involved in different regulatory mechanisms at both transcriptional and post-transcriptional levels, playing key roles in both physiological and tumorigenic processes [102, 103]. LncRNAs can be post-transcriptionally polyadenylated, spliced and capped. Additionally, a number of modifications can be found on lncRNA, including m^6^A, m^5^C, m^1^A, A-to-I editing and Ψ [104]. Recently, transcriptome-wide mapping of these RNA modifications allowed an extensive characterization of the lncRNA-epitranscriptome. Although further investigations are needed to decipher more specific regulatory roles of lncRNA transcripts, several studies demonstrated that RNA modifications affect metabolism, structure, RNA-protein interaction and cellular sub-localization of different lncRNAs, especially in cancer cells [105].

m^6^A is the most characterized modification on lncRNAs (Figure 2), first identified on the X-Inactive Specific Transcripts (*XIST*), the Metastasis Associated Lung Adenocarcinoma Transcript 1 (*MALAT1*) and the HOX Transcript Antisense RNA (*HOTAIR*) [3]. A recent study highlighted how m^6^A modification of *XIST,* mediated by METTL3/METTL14, induces its degradation, as expected from the known effects of this modification on mRNA. Loss of METTL14 in human colorectal cancers correlates with high levels of *XIST* and poor patient survival [106] (Table 2).

The m^6^A demethylases ALKBH5 activity on lncRNA seems to have opposite roles in different cancer types. Demethylation of *KCNK15-AS1* [107] leads to increased stability and inhibition of tumour progression. In contrast, ALKBH5 activity promotes invasion and metastasis of gastric cancer cells by increasing NEAT1 stability [108].

While m^6^A generally decreases the stability of transcripts, the opposite effect on a specific subset of RNAs was previously reported [109]. lncRNA *RP11* is upregulated by the overexpression of METTL3 [110], probably through its nuclear retention when modified. In turn, *RP11* stabilizes ZEB1 protein [110], a known pro-tumorigenic transcription factor [111] (Table 2). In nasopharyngeal cancer, *FAM225A* is highly modified and acts as a sponge for the tumour-suppressive miRNAs *miR-590-3p* and *miR-1275* [112]. It is not clear how this increased m^6^A modification occurs since alterations of m^6^A writers and erasers are not reported in this study.

Beyond the divergent regulation of lncRNAs stability, m^6^A modification could also influence the structural conformation of lncRNA molecules. METTL16 modifies the triple helix structure of *MALAT1*, essential for the interaction with its protein partners [113, 114]. Given that *MALAT1* plays a role in key cellular processes (such as alternative splicing and transcriptional regulation) and its expression is altered in several cancer types, further investigations may reveal the role of m^6^A modification of *MALAT1* in cancer cells. Similarly, many m^6^A sites were identified on *HOTAIR* transcripts [3]. Importantly, one of the m^6^A sites was shown to regulate *HOTAIR* recruitment to chromatin and promote breast cancer cells proliferation [115].

Similar to other systems, both the biological role and molecular mechanisms mediated by m^6^A on lncRNA is highly heterogeneous and cancer type dependent. Despite showing insight into the role of m^6^A in lncRNAs, one major problem with these studies is that they generally fail to address whether the effect of m^6^A modification of lncRNA is indeed responsible for the observed cancer phenotypes. Considering that m^6^A can affect the stability and translation of thousands of coding and non-coding transcripts, further studies are required to determine the specific effect of lncRNA modifications.

To date, different transcriptome-wide studies have mapped novel m^5^C sites on many lncRNAs [116–120] (Figure 2), including several involved in cancer. Despite this, the effect of this modification on lncRNA is still poorly understood. In oesophageal carcinoma, m^5^C is highly abundant on *NMR* lncRNA (also known as *LINC01672*), supporting its overexpression and promoting tumour progression [121] (Table 2). Overexpression of NSUN2 and m^5^C modification of *NMR* promote its stability. In turn, *NMR* upregulates the transcription of pro-metastatic factors such as MMP3 and MMP10.

The function and mechanism of Ψ on lncRNA and cancer progression remain to be elucidated, although many Ψ sites have been identified on different lncRNA transcripts, including *MALAT1, SRA1 and XIST,* [15, 122, 123] (Figure 2). To date, no specific role of Ψ in these RNAs was found in cancer cells, but further investigation might provide valuable evidence.

The telomerase RNA component (*TERC*) possesses highly conserved Ψ residues within a region essential for telomerase activity and TERT binding [44]. High expression of *TERC*, DKC1 and high levels of Ψ on telomerase RNA correlate with poor prognosis and malignant progression of lung [44] and prostate cancer [45]. DKC1 overexpression may be required for telomere homeostasis in these cancer types.

ADAR1-mediated A-to-I editing is widespread on *dsAlu* transcripts, which originated from transposable elements. Editing is used as a strategy to prevent interferon response in healthy cells [124, 125] (Figure 2). This mechanism is exploited by cancer cells, where the suppression of dsRNA by ADAR1 contributes to blunt cellular interferon response in cancer cells [126]. Importantly, Ishizuka and colleagues showed that inhibition of ADAR1 may be a viable strategy to sensitize melanoma cells to checkpoint blockade immunotherapy [126] (Table 2). Finally, A-to-I editing on the lncRNA prostate cancer antigen 3 (*PCA3*) enhanced its ability to bind and suppress the *PRUNE2* pre-mRNA, thus promoting cancer cell proliferation, adhesion and migration [127].

Overall, despite increasing evidence that lncRNAs are decorated with a number of modifications, their role in cancer is just starting to emerge and their clinical relevance is still uncertain. An important exception is A-to-I editing mediated by ADAR1 and its effect on dsRNA.

### Circular RNAs

Circular RNAs (circRNAs) comprise a large class of ncRNAs originating by backsplicing events, in which a downstream splice donor site is covalently linked to an upstream splice acceptor site [128]. Most circRNAs are expressed from known protein-coding genes and contain single or multiple exons, some of which are not included in the corresponding linear transcripts. Intronic sequences could be retained in the circular transcripts originating the so-called circular intronic RNAs (ciRNAs) [128]. Recent studies identified several functions of circRNAs in both physiological and pathological processes. circRNAs can directly act as miRNA sponge, proteins scaffold or decoy but can also be translated in a cap-independent manner [129].

Recently, two independent studies mapped m^6^A modification to thousands of human circRNA transcripts [130, 131] (Figure 2), picturing a selective methylome on these molecules with respect to their linear counterparts. Functionally, METTL3/14-induced m^6^A recruits the translational initiation factor eIF4G2 to the start codon of exons contained in circRNAs and, in turn, promotes their cap-independent translation [131]. Moreover, m^6^A-modified circRNAs showed distinct expression profiles comparing embryonic and cancer cells, suggesting a specific tumorigenic pattern [130]. Interestingly, m^6^A methylation is observed on a number of circRNAs originating from unmodified coding transcripts [130]. These results suggest the existence of a specific mechanism controlling m^6^A deposition on circRNAs. Furthermore, m^6^A is required to direct the backsplicing reaction, as demonstrated for *circZNF609* in rhabdomyosarcoma tumours, providing a link between m^6^A deposition and circRNA biogenesis [132]. *circZNF609* is also translated in a cap-independent manner and its expression correlates with the proliferative status of cells, pointing out its potential role in sustaining rhabdomyosarcoma cell growth, where proliferation predominates over differentiation [132].

A different function of m^6^A-modified circRNAs was discovered in colorectal cancer, where the overexpression of *circNSUN2* was identified in tumour tissues and serum samples from colorectal carcinoma patients with liver metastasis and predicts poor patient survival [133]. In this study, m^6^A modification of *circNSUN2* modulates its export from the nucleus to the cytoplasm, enhancing the stability of *HMGA2* mRNA by forming a *circNSUN2*/IGF2BP2/*HMGA2* RNA–protein ternary complex [133]. Finally, it was demonstrated that the m^6^A modification marks and signals ‘self’ circRNAs, whereas foreign and exogenous circRNAs are unmodified, triggering both innate and adaptive immune responses [134]. This evidence sets the ground for the inhibition of circRNA m^6^A modification as a strategy to trigger immune response to cancer cells.

The investigation of circRNAs epitranscriptomics is still at a very early stage: given the nature of circRNAs, most of the common detection methods are unable to detect their modifications and therefore dedicated epitranscriptomic studies will be required.

### Final remarks

The landscape of ncRNA in eukaryotic cells encompasses a great variety of subtypes and functions. ncRNAs are involved in all processes in living cells, from the most fundamental mechanisms of protein translation to the fine-tuning of gene expression and response to signalling and environmental cues. Therefore, it is not surprising that ncRNAs are implicated in the process of cell transformation and cancer progression on multiple levels.

RNA modifications add another layer of complexity to the non-coding transcriptome. They can directly influence the three-dimensional structure of RNA [19], affect their binding to proteins and other RNAs or regulate their turnover [11]. ncRNAs such as rRNAs and tRNAs are the most abundantly modified RNAs in human cells. Despite the dysregulation of single modifications within these subtypes does not generally compromise their functionality in normal physiology, it is likely that rRNA and tRNA modifications are required for fine-tuning translation. Cancer cells, constantly growing in stress conditions, may become ‘addicted’ to specific rRNA and tRNA modifications, specifically the ones regulating translation of key oncogenes and tumour suppressors.

Modification of miRNAs can affect multiple aspects of their function. Firstly, their complex post-transcriptional processing is prone to be tightly controlled and several modifications act at this level. Besides processing, modifications of miRNAs can influence their stability and impact mRNA targeting. In this way, alteration of miRNA modifications can have a simultaneous effect on the expression of a large number of coding transcripts. The case of A-to-I editing is peculiar since multiple reports show that editing of *miR-455* [95] and *miR-589-3p* [96] can specifically steer them away from their usual mRNA targets and redirect them to tumour suppressive transcripts.

Finally, lncRNAs are also subject to extensive modifications, but the functions of such modifications are still largely unknown. Importantly, lncRNAs mechanism of action *per se* is not generally well understood. Further studies will be required to elucidate the functional effects of modifications on lncRNAs.

Altogether the reported studies strongly support the central role of ncRNA epigenetics in cancer. RNA modifiers, being catalytically active enzymes, are ideal candidates as drug targets. Thus, the development of epitranscriptomic therapeutics will provide new strategies to modulate ncRNAs involved in cancer.

**Key Points**

- Chemical modifications of non-coding RNA are abundant and heterogeneous.
- Modifications of transfer RNA and ribosomal RNA have specific roles in cancer cells.
- Modifications of microRNA control gene expression programs in cancer.
- Epigenetic regulation of long non-coding RNA directly controls their functions.
- Enzymes responsible for non-coding RNA modifications show great therapeutic potential in cancer.

## Notes

**Valentina Miano** obtained her PhD in non-coding RNA biology at the University of Turin, where her research was dedicated to investigating the role of lncRNAs in breast cancer. She is now a Research Associate at the University of Cambridge working on RNA modifications in blood cancer.

**Azzurra Codino** obtained her PhD in stem cell biology at the University of Edinburgh in 2020. She is currently working as a postdoctoral fellow in developmental neurobiology of RNA at the Istituto Italiano di Tecnologia, in Genoa.

**Luca Pandolfini** was a postdoctoral fellow at the Gurdon Institute (University of Cambridge). He is now a Researcher in the Istituto Italiano di Tecnologia (Genoa) in the field of developmental neurobiology ofRNA.

**Isaia Barbieri** is a Junior Research Group Leader in the Department of Pathology at the University of Cambridge. His research is focused on the investigation of RNA epigenetic mechanisms in human cancers. His interest in RNA modification started during his postdoctoral training at the Gurdon Institute (University of Cambridge).
